# Effect of Rhesus D incompatibility on schizophrenia depends on offspring sex

**DOI:** 10.1016/j.schres.2008.06.022

**Published:** 2008-08-09

**Authors:** Christina G.S. Palmer, Erin Mallery, Joni A. Turunen, Hsin-Ju Hsieh, Leena Peltonen, Jouko Lonnqvist, J. Arthur Woodward, Janet S. Sinsheimer

**Affiliations:** a Department of Psychiatry & Biobehavioral Sciences, University of California, Los Angeles, CA, 90095, USA; b Department of Human Genetics, University of California, Los Angeles, CA, 90095, USA; c Department of Biostatistics, University of California, Los Angeles, CA, 90095, USA; d Institute for Molecular Medicine Finland FIMM and National Public Health Institute, 00251 Helsinki, Finland; e Genentech Corporation, San Francisco, CA, 94080, USA; f Department of Medical Genetics, University of Helsinki, 00251 Helsinki, Finland; g Program in Medical and Population Genetics, Broad Institute of Harvard and Massachusetts Institute of Technology, Cambridge, MA, 02142, USA; h Department of Mental Health and Alcohol Research, National Public Health Institute, 00251 Helsinki, Finland; i Department of Psychiatry, University of Helsinki, 00251 Helsinki, Finland; j School of Social Sciences, Humanities and Arts, University of California, Merced, CA, 95344, USA; k Department of Biomathematics, University of California, Los Angeles, CA, 90095 USA; l Wellcome Trust Sanger Institute, Wellcome Trust Genome Campus, Hinxton, Cambridge, CB10 1SA, UK

**Keywords:** Prenatal complications, Obstetric complications, Neurodevelopment, Hemolytic disease of the newborn, Maternal-fetal genotype incompatibility, Blood group incompatibility

## Abstract

Rhesus D incompatibility increases risk for schizophrenia, with some evidence that risk is limited to male offspring. The purpose of this study is to determine whether risk for schizophrenia due to Rhesus D incompatibility differs by offspring sex using a nuclear family-based candidate gene approach and a meta-analysis approach. The genetic study is based on a sample of 277 nuclear families with *RHD* genotype data on at least one parent and at least one child diagnosed with schizophrenia or related disorder. Meta-analysis inclusion criteria were (1) well-defined sample of schizophrenia patients with majority born before 1970, (2) Rhesus D incompatibility phenotype or genotype data available on mother and offspring, and by offspring sex. Two of ten studies, plus the current genetic study sample, fulfilled these criteria, for a total of 358 affected males and 226 affected females. The genetic study found that schizophrenia risk for incompatible males was significantly greater than for compatible offspring (*p* = 0.03), while risk for incompatible and compatible females was not significantly different (*p* = .32). Relative risks for incompatible males and females were not significantly different from each other. Meta-analysis using a larger number of affected males and females supports their difference. Taken together, these results provide further support that risk of schizophrenia due to Rhesus D incompatibility is limited to incompatible males, although a weak female incompatibility effect cannot be excluded. Sex differences during fetal neurodevelopment should be investigated to fully elucidate the etiology of schizophrenia.

## 1. Introduction

Schizophrenia is a debilitating disorder with a mixture of characteristic signs and symptoms, both positive and negative, affecting ~1% of the population (American Psychiatric Association, 1994). The positive symptoms include distortions in thought content (delusions), perception (hallucinations), language and thought process (disorganized speech), and self-monitoring of behavior (grossly disorganized or catatonic behavior). Negative symptoms include restrictions in the range and intensity of emotional expression (affective flattening), in the fluency and productivity of thought and speech (alogia), and in the initiation of goal-directed behavior (avolition) (American Psychiatric Association, 1994). Although the etiology of schizophrenia is unknown, it is likely a complex disorder involving genetic and environmental factors (Mueser and McGurk, 2004).

Sex differences in schizophrenia have been noted since Kraepelin found that dementia praecox occurred primarily in young men (Kraepelin, 1919/1971), and more recent investigations have revealed a number of sex differences in the characteristics of schizophrenia. Several recent studies have reported that males have a higher risk for developing schizophrenia compared to females. For example, Aleman et al. (2003) and Thorup et al. (2007) both find that the incident rate is higher in males than in females in ~1.4:1 ratio. Compared to females, males experience an earlier age at onset of schizophrenia (Castle and Murray, 1991; Mueser and McGurk, 2004; Thorup et al., 2007), have poorer premorbid social and intellectual functioning, poorer course and medication response, and greater structural brain abnormalities (Salem and Kring, 1998). Symptomatology also differs by sex, with males exhibiting more negative symptoms and females exhibiting more affective symptoms (Leung and Chue, 2000). Identification of sex-specific differences in schizophrenia is important because they may yield further clues about the etiology of this disorder, and this is the focus of the current paper.

Although the explanation for sex-specific differences is not yet known, some researchers suggest that male–female differences in schizophrenia may be explained through a neurodevelopmental perspective, where more males than females are likely to experience a form of the disease due to a neurodevelopmental anomaly resulting from early environmental influences (Castle and Murray, 1991). The neurodevelopmental hypothesis of schizophrenia is supported by numerous accounts of an association between obstetric complications (a form of early environmental influence) and risk of developing schizophrenia (Byrne et al., 2007; Cannon et al., 2000; Clarke et al., 2006; Geddes and Lawrie, 1995), which fall into three general categories: complications of pregnancy (bleeding, diabetes, rhesus incompatibility, preeclampsia), abnormal fetal growth (low birthweight, congenital malformations, reduced head circumference) and development, and complications of delivery (uterine atony, asphyxia, emergency Cesarean section) (Cannon et al., 2002). The role of the prenatal environment in schizophrenia is also supported by a recent meta-analysis of 12 twin studies which found that common or shared environment (which would most likely occur very early in life, i.e., prenatally) had a nontrivial contribution to the heritability of schizophrenia (~11%) (Sullivan et al., 2003).

Although the prenatal environment offers one plausible source for male–female differences in schizophrenia, there have been few detailed sex-specific studies and the results of such studies as yet are inconclusive. Some studies have documented an increased frequency of obstetric complications in pregnancies of males with schizophrenia (Byrne et al., 2000; Dalman et al., 1999; Hultman et al., 1999; O'Callaghan et al., 1992; Preti et al., 2000), others have documented an increased frequency of obstetric complications in pregnancies of females with schizophrenia (Verdoux and Bourgeois, 1993), and still other studies have concluded that there is no difference in rates of obstetric complications by sex of affected individuals (Byrne et al., 2007; Geddes et al., 1999; Verdoux et al., 1997). However, when we draw our attention to specific obstetric complications, such as Rhesus D incompatibility, there is evidence to suggest that there may be sex-specific differences related to risk for schizophrenia (Byrne et al., 2000; Hollister et al., 1996; Insel et al., 2005) and that is the focus of this paper.

The Rhesus D factor is a protein found on the surface of red blood cells (Guyton, 1981). The *RHD* locus [MIM 111680] (Online Mendelian Inheritance in Man) codes for the Rhesus D factor, and individuals are classified as Rhesus D positive or negative if the Rhesus D factor is present or absent on their red blood cells, respectively. Rhesus D status can be determined either genotypically or serologically (Wagner and Flegel, 2000; Westhoff, 2007). Rhesus D incompatibility is defined as a Rhesus D negative pregnant female with a Rhesus D positive fetus. Rhesus D incompatibility can result in maternal isoimmunization against fetal red blood cells and their subsequent hemolysis during pregnancy and in the neonatal period (Guyton, 1981). This process can lead to fetal and neonatal hypoxia and hyperbilirubinemia (Guyton, 1981), conditions that have been associated with schizophrenia (Amit and Brenner, 1993; Cannon, 1997; Cannon et al., 2000; Dalman et al., 2001; Hansen, 2000, 2001; Moises et al., 2002). The outcomes of Rhesus D incompatibility are variable (Plevyak and Carr, 2001) and Rhesus D hemolytic disease of the newborn (HDN) is a term used to describe the clinically measurable adverse outcomes of Rhesus D incompatibility in the neonatal period, e.g., anemia, hyperbilirubinemia, which in their most severe manifestation can lead to hydrops fetalis, kernicterus, and death (Bowman, 1998; Plevyak and Carr, 2001). The maternal immune response depends on previous exposure to fetal Rhesus D positive antigens during pregnancy or delivery, and the first incompatible pregnancy generally is not at risk for HDN (Guyton, 1981). However, among Rhesus negative women who have been isoimmunized, all subsequent incompatible pregnancies are at risk for HDN. Prophylaxis against maternal isoimmunization became available in North America in 1968 (Bowman, 1998) and in Finland in 1969 (Eklund and Nevanlinna, 1986), and has since significantly reduced morbidity and mortality of Rhesus D incompatibility worldwide.

There have been eight studies (Byrne et al., 2000; Hollister and Kohler, 2001; Hollister et al., 1996; Insel et al., 2005; Kendell et al., 2000; Kraft et al., 2004; Palmer et al., 2002; Sacker et al., 1995) and 2 meta-analyses (Cannon et al., 2002; Geddes et al., 1999) to directly or indirectly examine Rhesus D incompatibility as a risk factor for schizophrenia (briefly summarized in Table 1). The indirect studies tend to represent the earlier studies of Rhesus D incompatibility and schizophrenia, and are based on maternal recall or information from labor and birth records ascertained retrospectively or prospectively. Direct studies of Rhesus D incompatibility and schizophrenia are more recent and are based on serologic or genotype data from medical records or typed specifically for the study. Although the studies vary in the use of direct and indirect measures of Rhesus D incompatibility, collectively there is compelling evidence that Rhesus D incompatibility is a risk factor for schizophrenia (see Table 1). Moreover, in those studies that examined male–female differences in risk (Byrne et al., 2000; Hollister et al., 1996; Insel et al., 2005; Kendell et al., 2000; Sacker et al., 1995), there is evidence from several to suggest that the risk may be limited to male offspring, with relative risk ranging from 2.02–3.32. However, due to small subgroup sample sizes, particularly for affected female offspring, and variability in the use of direct and indirect measures of Rhesus D incompatibility, these sex-specific results remain inconclusive.

In this paper, we investigate if the effect of Rhesus D incompatibility on schizophrenia is limited to male offspring using a direct measure of Rhesus D incompatibility and two methodological approaches. The first methodological approach uses a direct measure of Rhesus D incompatibility in a candidate gene study of 277 nuclear families—the 181 nuclear families with at least one child diagnosed with schizophrenia or a related disorder previously analyzed (Kraft et al., 2004) plus an additional 96 nuclear families. The second approach uses meta-analysis to combine the results of the current analysis with sex-specific information on Rhesus D incompatibility available in two earlier studies that classified Rhesus D incompatibility using a direct measure (Hollister et al., 1996; Insel et al., 2005).

## 2. Materials and methods

### 2.1. Subjects

Subjects were ascertained from a larger well-described and characterized Finnish sample of schizophrenia patients and their families (Ekelund et al., 2001; Ekelund et al., 2000; Hovatta et al., 1999; Paunio et al., 2001). Briefly, probands, born between 1940 and 1969, were initially identified through nationwide population and health registries, and first-degree relatives were contacted with permission from the proband. Informed consent was obtained from all subjects. DSM-IV (American Psychiatric Association, 1994) best estimate psychiatric diagnoses on probands and their relatives were made by two independent psychiatrists or psychiatric residents from information recorded in inpatient and outpatient medical records. The research was approved by the Ministry of Social Affairs and Health (Finland) and the institutional review board at the University of California, Los Angeles (USA). Individuals were classified as affected if they were diagnosed with schizophrenia, schizoaffective psychosis disorder, or a schizophrenia spectrum disorder (paranoid personality, schizoid personality, or schizotypy). Independent nuclear families with at least one genotyped parent and at least one genotyped affected child were used in the analysis.

The study sample is comprised of 277 nuclear families with the number of children ranging from 1 to 7 per family. Of the 677 children, 505 were classified as affected (303 affected males, 202 affected females). Genotype data were available on both parents in 132 families, while they were available only for the mother in 107 families, and only for the father in 38 families. 58 affected offspring were determined to be either unambiguously Rhesus D incompatible with their mothers (24 males, 14 females) because genotype information was available on mother and offspring, or potentially incompatible with their mothers (15 males, 5 females) in the case of heterozygous offspring with Rhesus D positive fathers, missing maternal genotypes, and sibling genotypes that are consistent with a Rhesus D negative mother. In 21 independent families the unambiguously or potentially incompatible affected off-spring were male, in 6 families the incompatible offspring were female, and in 10 families both male and female incompatible offspring were present. Birth years for the offspring ranged from 1923–1976, with only 12 offspring born in or after 1970, the era when prophylaxis against maternal isoimmunization became widely used in Finland (Eklund and Nevanlinna, 1986). However, none of these 12 individuals resulted from incompatible pregnancies and so it is unlikely that the prophylaxis was used or was even necessary.

### 2.2. Genotyping methods

A standard procedure was used to extract DNA from EDTA blood (Blin and Stafford, 1976) and a PCR-RFLP method was used to identify all three *RHD* genotypes (D/D, D/d, and d/d) (Wagner and Flegel, 2000). PCR was performed using primers rez7 (5′-CCTGTCCCCATGATTCAGTTACC-3') and rnb1 (5'-CCTTTTTTTGTTTGTTTTTGGCGGTGC-3′) and the expand high fidelity PCR system (DyNAzyme™ EXT DNA Polymerase). Annealing was at 65 °C and extension was for 3 min at 72 °C. PCR products were digested with PstI for 3 h at 37 °C, and fragments were visualized using a 1.5% agarose gel. Genotyping was performed blind to diagnostic status and sex. Genotype data were checked for genotyping errors using Mendel 6.0 (Lange et al., 2001) resulting in the exclusion of 5 families with genotyping error probabilities ≥0.50. There was no evidence for violation of Hardy–Weinberg equilibrium among the founders (*p*=0.6). Among the 409 genotyped parents, 52 were dd, 189 were Dd, and 168 were DD; the frequency of the D and d alleles were 64.2% and 35.8%, respectively. These data are comparable to the frequency of Rhesus D negative (dd) individuals in Finland (~12%) (Finnish Red Cross), and the frequency of the d allele in the Finnish population (~34%) (Harvey et al., 1983).

### 2.3. Statistical analyses

For the purposes of these analyses, the *RHD* gene is treated as diallelic, with D denoting all polymorphisms that lead to protein expression and d alleles denoting all polymorphisms that do not. An individual who inherits two d alleles (dd) is Rhesus D negative while those who inherit at least one D allele (Dd or DD) are Rhesus D positive. Rhesus D incompatibility occurs when the mother is dd and the child is Dd. To test for association of Rhesus D incompatibility and schizophrenia, we used a conditional log-linear model (Kraft et al., 2004) which is a multi-sibling extension of the maternal-fetal genotype incompatibility (MFG) test (Sinsheimer et al., 2003).

The parental and offspring genotype data are modeled as conditional on the offspring phenotypes. Each family is independent so Eq. (1) denotes the total likelihood from *N* families

(1) $$\overset{N}{\underset{i=1}{\Pi }}\mathrm{Pr}\left(G_{c}^{\left(i\right)},G_{p}^{\left(i\right)}|D^{\left(i\right)}\right)$$

The index *i*=1,…, *N*, denotes family,$G_{c}^{\left(i\right)}=\left(G_{\mathrm{cl}}^{\left(i\right)},\ldots G_{{\mathrm{cn}}_{i}}^{\left(i\right)}\right)$ denote the genotypes of the *n*_*i*_ offspring, in which *K_i_* are affected;$G_{p}^{\left(i\right)}=\left(G_{m}^{\left(i\right)},G_{f}^{\left(i\right)}\right)$ denote parental genotypes, in which $G_{m}^{\left(i\right)}$ and $G_{f}^{\left(i\right)}$ are maternal and paternal genotypes, respectively and $D^{\left(i\right)}=\left(D_{1}^{\left(i\right)},\ldots D_{n_{i}}^{\left(i\right)}\right)$ denotes the affection status for each offspring. Unaffected offspring are treated as phenotype unknown (missing) since the prevalence of schizophrenia is relatively low (Hsieh et al., 2006a). In assuming that affected siblings' phenotypes are independent given their parental genotypes, we can express likelihood (Eq. (1)) as a function of the offspring's penetrance, Mendelian transmission probabilities, and population mating type frequencies (Eq. (2)) using Bayes theorem,

(2) $$\begin{array}{cc} & \underset{i}{\Pi }\mathrm{Pr}\left(G_{c}^{\left(i\right)},G_{p}^{\left(i\right)}|D^{\left(i\right)}\right)\\ & \;=\underset{i}{\Pi }\frac{\mathrm{Pr}\left(G_{c}^{\left(i\right)}|G_{p}^{\left(i\right)}\right)\mathrm{Pr}\left(G_{p}^{\left(i\right)}|{\mathrm{MT}}_{i}\right)\psi_{{\mathrm{MT}}_{i}}\underset{k=1}{\Pi }\mathrm{Pr}\left(D_{\mathrm{ck}}^{\left(i\right)}|G_{\mathrm{ck}}^{\left(i\right)},G_{m}^{\left(i\right)}\right)}{\mathrm{Pr}\left(D^{\left(i\right)}\right)}, \end{array}$$,

where *MT_i_* indexes the parental mating type and the nuisance parameters *ψ_MT_i__* are population mating-type frequencies. Our penetrance function $\mathrm{Pr}\left(D_{\mathrm{ck}}^{\left(i\right)}|G_{\mathrm{ck}}^{\left(i\right)},G_{m}^{\left(i\right)}\right)$, the probability of the offspring phenotype given the offspring genotype *and* maternal genotype, differs from the standard genetic penetrance function $\mathrm{Pr}\left(D_{\mathrm{ck}}^{\left(i\right)}|G_{\mathrm{ck}}^{\left(i\right)}\right)$, the probability of the offspring phenotype given the offspring genotype *only*. As in previous analyses of multiple affected siblings (Hsieh et al., 2007; Hsieh et al., 2006a; Kraft et al., 2004), we model the penetrance as a function of the relative risks of disease for offspring depending on their risk category. Based on the results of previous studies (Kraft et al., 2004; Palmer et al., 2002), we exclude child allelic effects. Incompatibility is defined as the combination of maternal genotype=dd (Rhesus D negative) and affected offspring genotype=Dd (Rhesus D positive); all remaining maternal-offspring genotype combinations are treated as non-risk. In our most general hypothesis, we include two variables, Minc, which equals 1 when a male offspring is incompatible with his mother and 0 when he is compatible, and Finc, which equals 1 when a female offspring is incompatible with mother and 0 when she is compatible. The penetrance function is then

(3) $$\mathrm{Pr}\left(D_{\mathrm{ck}}|G_{\mathrm{ck}},G_{m}\right)=\beta \mu_{\text{male}}^{I\left[\mathrm{Minc}\right]}\mu_{\text{female}}^{I\left[\mathrm{Finc}\right]}.$$.

*I*[.] is the indicator function and the parameter *β* denotes the population baseline disease incidence rate, which will cancel from (Eq. (2)). The parameter *μ*_male_ is the risk of schizophrenia for incompatible males and the parameter *μ*_female_ is the risk for incompatible females, both relative to any compatible offspring. Note that these relative risks are defined on the non-negative real line, and have null values of 1. Different models are generated for hypothesis testing by applying specific constraints to these parameters. As examples, placing *μ*_female_=1 assumes that the incompatibility effect is limited to males, while setting *μ*_male_=*μ*_female_ assumes that the incompatibility effect does not depend on offspring sex. Both of these models have one degree of freedom less than our most general model. The numerical maximization of the log-likelihood is better conditioned when we use the natural logarithm of the relative risks as parameters so we actually estimate ln (*μ*_male_) and ln(*μ*_female_); however we present the non-logged values throughout this paper.

As previously (Kraft et al., 2004; Palmer et al., 2002; Sinsheimer et al., 2003), the model is constructed without assumptions of Hardy–Weinberg equilibrium or random mating. Instead we make a more mild assumption of mating symmetry in the general population from which these families were ascertained. Under this assumption, there are six possible mating types. Data from families with incomplete parental genotypes can be included in (Eq. (2)) by assuming the genotypes are missing at random and summing over all possible parental genotypes (Hsieh et al., 2006b; Kraft et al., 2004). The MFG test also assumes that the probability of an affected individual surviving to clinical detection is independent of the maternal, paternal, and affected child's genotypes. However, the MFG test is insensitive to small violations of this assumption (Hsieh et al., 2006a), which is the case in these analyses because most children survive the sequelae of Rhesus D incompatibility even without prophylaxis (Plevyak and Carr, 2001).

The constrained log-likelihoods were maximized and maximum likelihood estimates of the parameters obtained using an application of the non-linear optimization program Search (Hsieh et al., 2006b; Lange, 1991). Likelihood ratio test (LRT) statistics and Akaike's information criterion (AIC) were used to compare different models, where smaller AIC values indicate a better fitting model (Burnham and Anderson, 1998). Where appropriate one-sided *p*-values are reported because prior evidence suggests that the Rhesus D incompatibility effect is deleterious (M. Cannon et al., 2002; Hollister et al., 1996; Insel et al., 2005; Palmer et al., 2002). The significance level for the LRTs is set at 0.05.

We also performed a meta-analysis using a fixed effects model (Fleiss, 1993) to estimate the Rhesus D incompatibility effect for males and females. For the meta-analysis, we used data generated from our study and data published in studies that fulfilled the following set of criteria: (1) well-defined sample of schizophrenia patients with the majority born before 1970, (2) Rhesus D incompatibility phenotype or genotype data available on mother and offspring, and by offspring sex.

The search strategies of a computerized MEDLINE search of English only studies published before November 2007 using search terms Rhesus D incompatibility, Rh incompatibility, or hemolytic disease of the newborn coupled with schizophrenia, cross-referencing of studies, and contacting researchers identified the 10 studies listed in Table 1. Two studies fulfilled our criteria for inclusion in the meta-analysis (Hollister et al., 1996; Insel et al., 2005). The remaining eight studies were not included either because their samples are a subset of the Finnish schizophrenia sample analyzed in the candidate gene study reported here (Kraft et al., 2004; Palmer et al., 2002) or because they used inferred indicators of maternal and offspring Rhesus D phenotypes which were either too broad for classifying Rhesus D incompatibility (Sacker et al., 1995) or too narrow for classifying Rhesus D incompatibility (Byrne et al., 2000; Cannon et al., 2002; Geddes et al., 1999; Hollister and Kohler, 2001; Kendell et al., 2000).

For the meta-analysis, a male and female relative risk, *μ*_male_ and *μ*_female_ respectively, was computed separately for the three studies. These relative risks were computed from the raw data available from the Finnish schizophrenia study sample and from the published data in Hollister et al. (1996) and Insel et al. (2005). To generate the largest possible sample size for analysis, we used data from all incompatible and compatible offspring in the Finnish schizophrenia study sample (303 affected males, 202 affected females) and the Hollister et al. sample (21 affected males, 5 affected females), and we used data from 2nd or later born incompatible and compatible offspring in the Insel et al. sample (34 affected males, 19 affected females) as a result of their definition of Rhesus D incompatibility. To make the analyses as comparable as possible between studies, we used relative risks that were unadjusted for censoring or covariates. This decision led to relative risks for the Insel et al. (2005) data that are slightly smaller than their published values. A homogeneity statistic, Q, was calculated to determine if there is evidence for a study-by-effect size interaction. This value follows a chi-square distribution with (# studies—1) degrees of freedom (Fleiss, 1993). Statistical evidence for heterogeneity would suggest that the studies should not be combined in a meta-analysis framework.

## 3. Results

### 3.1. Candidate gene study

First, we performed a series of analyses in order to test the hypothesis that the effect of Rhesus D incompatibility on schizophrenia is limited to males in our sample. Using the MFG test we estimated the parameters of a fully unconstrained model, i.e., one that estimates the male and female incompatibility effects separately (Table 2, Model 1). Inspection of Model 1 reveals that the estimate for the male incompatibility effect [*μ*_male_=1.44, 90% CI: 1.05–1.98] is higher than the female incompatibility effect [*μ*_female_=1.12, 90% CI: 0.74–1.70]. In order to test the hypothesis that *μ*_male_>1, we compared the fit of Model 1 to the fit of a model where *μ*_male_ is constrained to the null value of 1 (Table 2, Model 2). This comparison yielded *χ*^2^ (1*df*)=3.21, *p*=0.036 (one-sided), leading us to reject the null hypothesis of *μ*_male_=1 in favor of the alternative hypothesis that there is a positive male incompatibility effect. The model that constrains the female effect to one but allowsfor a male effect [*μ*_male_=1.44, 90% CI: 1.05–1.98] (Table 2, Model 3) had the smallest AIC of the models we examined (AIC=1681.2), providing further support that Rhesus D incompatibility is a risk factor for schizophrenia only in males. Table 3 provides the counts of parental genotype combinations underlying Model 3 for the 277 families in order to get a better feel for the data. The counts are fractional because mother–child and father–child dyads are probabilistically apportioned in an unbiased manner conditional on the possible mating types. Under the assumption of mating symmetry in the population and the null that incompatibility does not matter to risk, there should be about equal numbers of Rhesus D negative mothers and Rhesus D positive fathers as families with Rhesus D positive mothers and Rhesus D negative fathers. By contrast, Table 3 demonstrates an asymmetry in those counts and this finding is consistent with the alternative hypothesis that Rhesus D incompatibility is a risk factor for schizophrenia in males. Table 3 also reveals an even greater asymmetry between the ddmother/DD-father families versus the DD-mother/dd-father families, which is consistent with every fetus conceived by the former type of couple being subject to incompatibility, whereas no fetus conceived by the latter type would be incompatible.

In order to test the hypothesis that *μ*_female_>1, we compared the fit of Model 1 to the fit of a model where *μ*_female_ is constrained to the null value of 1 (Table 2, Model 3). This comparison yielded *χ*^2^ (1*df*)=0.220, *p*=0.319 (one-sided), consistent with the hypothesis that there is no female incompatibility effect. To test the sex independent model originally proposed (Palmer et al., 2002), we then directly tested the hypothesis that the incompatibility effects are independent of the sex of the offspring by comparing the fit of Model 1 to a model that assumes that the male and female incompatibility effects are equal [*μ*_male_=*μ*_female_, Table 2, Model 4]. This comparison did not lead to a rejection of the null hypothesis (*χ*^2^ (1*df*)=0.620, *p*=0.431 [two-sided]) and thus fails to provide additional support for the hypothesis that the incompatibility effect is exclusively for male offspring.

### 3.2. Meta-analysis

**Fig. 1:**
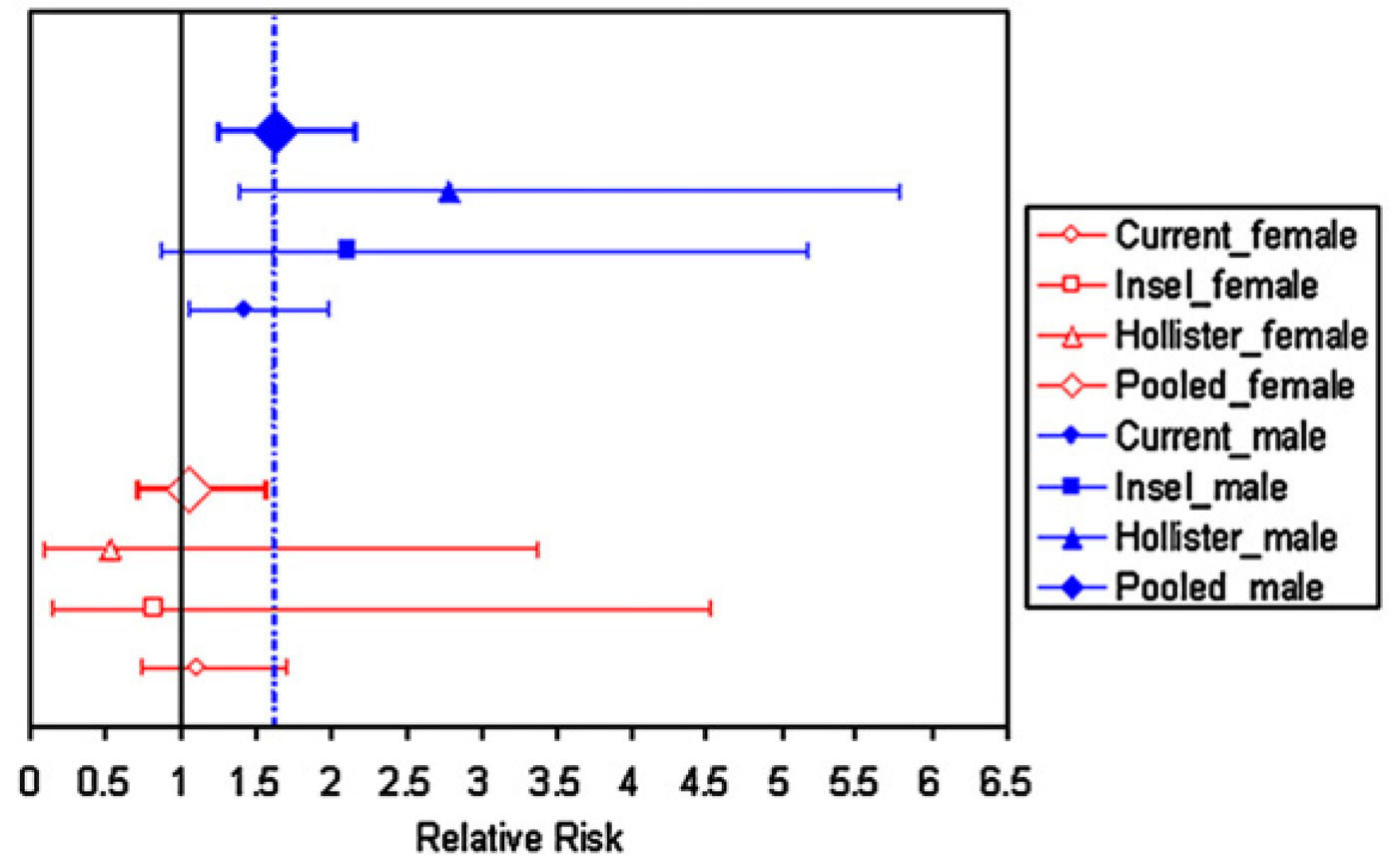
Individual study and pooled sex-specific estimates of Rhesus D incompatibility relative risk for schizophrenia. Filled symbols denote estimates for incompatible males; open symbols denote estimates for incompatible females. Diamond=estimate derived from meta-analysis; Triangle=estimate derived from data published in Hollister et al. (1996); Square=estimate derived from data published in Insel et al. (2005); Circle=estimate derived from current candidate gene study. Solid vertical line denotes null value and dashed vertical line denotes meta-analysis estimate for incompatible males.

We then performed a meta-analysis of three studies to estimate sex-specific Rhesus D incompatibility risks for schizophrenia. There was no evidence of heterogeneity across the three studies (*Q* statistic for male offspring data=2.07, *p*=0.356; *Q* statistic for female offspring data=0.485, *p*=0.785) indicating that pooling their results is justified (Fleiss, 1993). The pooled estimate of *μ*_male_=1.64 with 90% CI 1.25–2.17 and the pooled estimate of *μ*_female_=1.07, with 90% CI 0.72–1.58. The 90% CI for *μ*_male_ does not cover the null value of one, supporting an incompatibility effect in male offspring; while the 90% CI for *μ*_female_ includes the null value, providing additional support for an incompatibility effect that is limited to male offspring. Fig. 1 displays the study-specific estimates of *μ*_male_, *μ*_female_, and their upper and lower 90% CI values. In each individual study *μ*_male_ > *μ*_female_ which is consistent with the results of the meta-analysis.

## 4. Discussion

We examined Rhesus D incompatibility as a sex-dependent risk factor for schizophrenia using a candidate gene approach in a large sample and by performing a meta-analysis of the sex-specific incompatibility effects produced by datasets from three studies. The candidate gene approach yielded several lines of evidence to support a male incompatibility effect. First, we rejected the null hypothesis of no male incompatibility effect when comparing a model that estimated the sex-specific incompatibility effects to a model that constrained the male incompatibility effect to its null value; whereas we failed to reject the null hypothesis of no female incompatibility effect with an analogous model comparison. Second, we demonstrated that a model that constrains the female effect to one but allows for a male effect had the best fit by AIC of the models we examined, further supporting that Rhesus D incompatibility is a risk factor for schizophrenia only in males. In our sample, the relative risk of schizophrenia in male Rhesus D incompatible pregnancies was greater than for female incompatible pregnancies (1.44 and 1.12, respectively). However, when we compared a model with these estimates of the male and female incompatibility effects to a model that constrained the sex-specific effects to be equal, we found that the two models were not statistically different, thus failing to provide additional support for the hypothesis that the incompatibility effect is exclusively for male offspring.

By combining three datasets in the meta-analysis, we increased the sample size for affected males to 358 and affected females to 226 (a 12–18% increase from the Finnish sample). The results of our meta-analysis yielded the strongest evidence thus far that the incompatibility effect is limited to male offspring. The point estimate of the pooled female incompatibility effect is not only smaller than that for males [*μ*_female_ = 1.07, 90% CI 0.72–1.58; *μ*_male_ = 1.64, 90% CI 1.25–2.17], it also does not fall within the 90% CI for the male incompatibility effect, and it is contained within a 90% CI that includes the null value. Furthermore, the 90% CI for the male incompatibility effect does not cover the null value of one and the effect estimate for males does not fall within the 90% CI for the female effect.

The results of our candidate gene analysis and meta-analysis suggest that the effect of Rhesus D incompatibility on the development of schizophrenia is limited to male offspring. However, because the sample size for females was ~35% less than that of the males, our study may have lacked sufficient statistical power to detect a small female effect. On the other hand, the estimated pooled relative risk for females in the meta-analysis is 1.07, which is very small in magnitude, leaving in question its substantive meaningevenif found to be statistically significant in a larger sample. A null or very small female effect relative to males is an important finding that allows for hypothesis generation and testing to determine why schizophrenia effects of Rhesus D incompatibility are so much greater for male offspring compared to female offspring.

One hypothesis is that male fetuses are at greater risk because clinical manifestations of Rhesus D incompatibility are much more likely to occur in pregnancies with male fetuses than with female fetuses. Although there is some literature that suggests that Rhesus D negative mothers are more likely to be *immunized* by male fetuses than female fetuses (Renkonen and Timonen, 1967; Woodrow and Donohoe, 1968), there is no evidence that its related condition of Rhesus D incompatibility HDN is more likely to occur in subsequent pregnancies with male fetuses than with female fetuses. Hence, this is unlikely to explain male–female differences in the incompatibility effect.

A related hypothesis is that clinical manifestations of Rhesus D incompatibility must surpass a threshold of severity before they increase risk for schizophrenia, and male fetuses are more likely to experience sequelae that surpass this threshold than female fetuses. This hypothesis predicts that the clinical manifestations of Rhesus D incompatibility would be more severe in pregnancies with male fetuses than with female fetuses. Evidence to support this hypothesis comes from Ulm et al. (1999),where they found that compared to incompatible female fetuses, incompatible male fetuses had a significantly lower relative hemoglobin and hematocrit at their initial intravenous transfusion, required more intravenous transfusions, and were more likely to develop hydrops. In addition, more male fetuses than female fetuses died in utero or during the neonatal period (Ulm et al., 1999). An implication of this hypothesis is that specific schizophrenia-effects of Rhesus D incompatibility (i.e., hypoxia, hyperbilirubinemia) can affect female fetuses but that females are less likely to surpassthe threshold compared to male fetuses.

A third hypothesis is that the timing of manifestations of Rhesus D incompatibility during gestation coincides with sex-specific maturational events such that the developing male brain is more vulnerable to effects of Rhesus D incompatibility than the developing female brain. Research supports differences in brain maturational rates in males and females (Cahill, 2006), where the pace of cerebral development is slower in males than females (Castle and Murray, 1991). Hence, the slower-maturing brains of males may be vulnerable to environmental insults for a longer period of time than the more quickly maturing brains of females (Castle and Murray, 1991). Placental transfer of maternal anti-D antibodies has been observed as early as the 18th week of gestation (Adinolfi, 1985) and evidence of hemolytic disease in the fetus has been demonstrated in the second trimester (Mollison, 1993), providing a timeframe for considering the effects of Rhesus D incompatibility. Of note, male fetuses have been found to require intravenous transfusion at an earlier gestational age than female fetuses (male median gestation age 24.5 weeks, range 18–35 weeks; female median gestational age 31.0 weeks, range 22–36 weeks) (Ulm et al., 1999), suggesting the possibility of a sex-dependent vulnerability to the timing of Rhesus D incompatible effects, with male fetuses experiencing an earlier and longer window of vulnerability during neurodevelopment than female fetuses. An implication of this hypothesis is that males and females may be equally vulnerable to the specific effects produced by Rhesus D incompatibility, but that these effects must occur at sex-dependent times during development. If this is the case, it would mean that female fetuses may be at increased risk for schizophrenia when subject to obstetric complications that produce the same downstream effects as HDN, e.g., hypoxia, hyperbilirubinemia, but that these effects must occur earlier in gestation to increase risk for schizophrenia in females.

A fourth hypothesis is that male fetuses are at greater risk because male developing brains are more vulnerable to one or both specific effects of the clinical manifestations of Rhesus D incompatibility, i.e., hypoxia, hyperbilirubinemia, than female developing brains. Data bearing on this hypothesis are conflicting with some studies finding higher rates of hypoxic-related obstetric complications in males with schizophrenia (Hultman et al., 1999), and others finding higher rates in females with schizophrenia than in males with schizophrenia (Goldstein et al., 2000).

Our findings suggest that the effects of Rhesus D incompatibility, and hence, adverse prenatal environments, on the development of schizophrenia may depend on offspring sex. To further bolster this conclusion, one would expect to observe an increased risk among 2nd or later born incompatible males, but not for females. However, because our datasets were not conducive for jointly analyzing the effect of prior Rhesus D incompatibility exposure and offspring sex in either the candidate gene or meta-analysis approach (due to the relatively small sample sizes of incompatible affected offspring and other study design limitations), we are unable to provide this additional piece of evidence. Nevertheless, our sex-dependent finding is particularly interesting in light of recent work demonstrating that HLA-B matching as a schizophrenia risk factor is limited to females (Palmer et al., 2006). Not only do these findings suggest that sex differences during fetal neurodevelopment should be investigated to fully elucidate the etiology of schizophrenia, but they allow investigators to generate several testable hypotheses regarding the effects of the prenatal environment on schizophrenia risk which may lead to further understanding of the basic processes underlying schizophrenia and preventative strategies as well.

## Figures and Tables

**Table 1:** Description of studies examining Rhesus D incompatibility as a schizophrenia risk factor

Study	Birth years (cases)	Measure of Rhesus D incompatibility	Rhesus D incompatibility effect	Sex-specific Rhesus D incompatibility effect
Hollister et al. (1996)	1959–1961	Rhesus D serotypes from medical records on mother and child	RR=1.99 (*p*=0.12)	All males: RR=2.78 (*p*=0.03) 2nd or later born males: RR=3.32 (*p*=0.05) All Females: RR=0.53 (*p*=1.0) 2nd or later born females: not examined due to small sample size
Insel et al. (2005)	1959–1967	Rhesus D serotypes from blood samples collected from mother and child at delivery	aRR_adj_=1.80 (95% CI 0.71–4.58)	2nd or later born males: RR=2.02 (95% CI 0.71–5.79) RR_adj_=2.37 (95% CI 0.82–6.86) 2nd or later born females: RR=0.90 (95% CI 0.12–6.74) RR_adj_=0.93 (95% CI 0.12–7.01)
Palmer et al. (2002)	1940–1969	Rhesus D genotypes from blood samples collected from mother and child	RR=2.26 (*p*=0.027)	Not examined
Kraft et al. (2004) b	1940–1969	Rhesus D genotypes from blood samples collected from mother and child	2nd or later born offspring RR=1.7 (*p*=0.014)	Not examined
Sacker et al. (1995)	1958	Mother noted as Rhesus negative in medical record	OR=1.79 (*p*=0.05)	Not examined
Kendell et al. (2000)	1971–1974	Presence of Rhesus D antibodies noted in medical record	OR=6.0 (*p*=0.10)	Reported as non-significant
Byrne et al. (2000)	Before 1972^c^	Not explicit	Not reported	Males: 1:255 cases to 0:256 controls (sample too small to compute stable estimates) Females: 2:173 cases to 2:173 controls
Hollister and Kohler (2001)	1955–1967	Evidence of hemolytic disease of the newborn in medical record	OR=2.0 (*p*=0.05)	Not examined
Geddes and Lawrie (1995) (meta-analysis)	Before 1976^d^	Evidence of hemolytic disease of the newborn in medical record or by maternal recall	OR=1.4 (*p*=0.49)	Reported as non-significant
Cannon et al. (2002) (meta-analysis of Sacker et al., 1995; Kendell et al., 2000; Byrne et al., 2000)	~1958–1974	Mother noted as Rhesus negative in medical record; Presence of Rhesus D antibodies noted in medical record; or not explicit	OR=2.0 (*p*=0.05)	Not examined

a RR_adj_ are adjusted for maternal age and ethnicity.

b Contains same nuclear families analyzed in Palmer et al., 2002, but expanded to include all genotyped affected and unaffected offspring.

c Cases most likely born before 1972 because they were diagnosed with schizophrenia between 1972 and 1992.

d Meta-analysis of studies published between 1990 and 1996, so cases likely born before 1976.

**Table 2:** Evaluation of four different models of risk due to Rhesus D incompatibility

Model	*μ*_male_ a (90% CI)	*μ*_female_ b (90% CI)	Log likelihood	AIC^c^
1	1.44 (1.05, 1.98)	1.12 (0.74, 1.70)	−834.51	1683.0
2	=1	1.14 (0.76, 1.71)	−836.12	1684.2
3	1.44 (1.05, 1.98)	=1	−834.62	1681.2
4	1.30 (1.01, 1.67)	=*μ*male	−834.82	1681.6

a *μ*_male_=the relative risk for male incompatible offspring.

b *μ*_female_=the relative risk for female incompatible offspring.

c AIC=Akaike's Information Criterion (smaller is better).

**Table 3:** Parental genotype combinations under model where Rhesus D incompatibility effect is limited to male offspring

Father	Mother		
	dd	Dd	DD
dd	11.54	12.06	7.85
Dd	15.17	71.27	45.33
DD	12.07	40.16	61.55

## References

[R1] Adinolfi M (1985). The development of the human blood-CSF-brain barrier. Dev. Med. Child. Neurol.

[R2] Aleman A, Kahn RS, Selten JP (2003). Sex differences in the risk of schizophrenia. Arch. Gen. Psychiatry.

[R3] American Psychiatric Association (1994). Diagnostic and Statistical Manual.

[R4] Amit Y, Brenner T (1993). Age-dependent sensitivity of cultured rat glial cells to bilirubin toxicity. Exp. Neurol.

[R5] Blin N, Stafford DW (1976). A general method for isolation of high molecular weight DNA from eukaryotes. Nucleic Acids Res.

[R6] Bowman JM (1998). RhD hemolytic disease of the newborn. N. Engl. J. Med.

[R7] Burnham K, Anderson D (1998). Model Selection and Inference: A Practical Information-Theoretic Approach.

[R8] Byrne M, Browne R, Mulryan N, Scully A, Morris M, Kinsella A, Takei N, McNeil T, Walsh D, O'Callaghan E (2000). Labour and delivery complications and schizophrenia. Br. J. Psychiatry.

[R9] Byrne M, Agerbo E, Bennedsen B, Eaton WW, Mortensen PB (2007). Obstetric conditions and risk of first admission with schizophrenia: a Danish national register based study. Schizophr. Res.

[R10] Cahill L (2006). Why sex matters for neuroscience. Nat. Rev. Neurosci.

[R11] Cannon TD (1997). On the nature and mechanisms of obstetric influences in schizophrenia: a review and synthesis of epidemio-logical studies. Int. Rev. Psychiatry.

[R12] Cannon TD, Rosso IM, Hollister JM, Bearden CE, Sanchez LE, Hadley T (2000). A prospective cohort study of genetic and perinatal influences in the etiology of schizophrenia. Schizophr. Bull.

[R13] Cannon M, Jones P, Murray R (2002). Obstetric complications and schizophrenia: historical and meta-analytic review. Am. J. Psychiatry.

[R14] Castle DJ, Murray RM (1991). The neurodevelopmental basis of sex differences in schizophrenia. Psychol. Med.

[R15] Clarke MC, Harley M, Cannon M (2006). The role of obstetric events in schizophrenia. Schizophr. Bull.

[R16] Dalman C, Allebeck P, Cullberg J, Grunewald C, Koster M (1999). Obstetric complications and the risk of schizophrenia: a longitudinal study of a national birth cohort. Arch. Gen. Psychiatry.

[R17] Dalman C, Hollie VT, David AS, Gentz J, Lewis G, Allebeck P (2001). Signs of asphyxia at birth and risk of schizophrenia. Br. J. Psychiatry.

[R18] Ekelund J, Lichtermann D, Hovatta I, Ellonen P, Suvisaari J, Terwilliger JD, Juvonen H, Varilo T, Arajarvi R, Kokko-Sahin M-L, Lonnqvist J, Peltonen L (2000). Genome-wide scan for schizophrenia in the Finnish population: evidence for a locus on chromosome 7q22. Hum. Mol. Genet.

[R19] Ekelund J, Hovatta I, Parker A, Paunio T, Varilo T, Martin R, Suhonen J, Ellonen P, Chan G, Sinsheimer JS, Sobel E, Juvonen H, Arajarvi R, Partonen T, Suvisaari J, Lonnqvist J, Meyer J, Peltonen L (2001). Chromosome 1 loci in Finnish schizophrenia families. Hum. Mol. Genet.

[R20] Eklund J, Nevanlinna HR (1986). Perinatal mortality from Rh(D) hemolytic disease in Finland, 1975–1984. Acta Obstet. Gynecol. Scand.

[R21] Finnish Red Cross http://www.veripalvelu.fi/uploads/pzqoiyv09.jpg.

[R22] Fleiss JL (1993). The statistical basis of meta-analysis. Stat. Methods Med. Res.

[R23] Geddes JR, Lawrie SM (1995). Obstetric complications and schizophrenia: a meta-analysis. Br. J. Psychiatry.

[R24] Geddes JR, Verdoux H, Takei N, Lawrie SM, Bovet P, Eagles JM, Heun R, McCreadie RG, McNeil TF, O'Callaghan E, Stober G, Willinger U, Murray RM (1999). Schizophrenia and complications of pregnancy and labor: an individual patient data meta-analysis. Schizophr. Bull.

[R25] Goldstein JM, Seidman LJ, Buka SL, Horton NJ, Donatelli JL, Rieder RO, Tsuang MT (2000). Impact of genetic vulnerability and hypoxia on overall intelligence by age 7 in offspring at high risk for schizophrenia compared with affective psychoses. Schizophr. Bull.

[R26] Guyton AC (1981). Textbook of Medical Physiology.

[R27] Hansen TW (2000). Bilirubin exidation in brain. Mol. Genet. Metab.

[R28] Hansen TW (2001). Bilirubin brain toxicity. J. Perinatol.

[R29] Harvey RG, Tills D, Warlow A, Kopec AC, Domaniewska-Sobczak K, Suter D, Lord JM (1983). Genetic affinities of the Balts: a study of blood groups, serum proteins and enzymes of Lithuanians in the United Kingdom. R. Anthropol. Inst. G.B. Irel.

[R30] Hollister JM, Kohler C (2001). Schizophrenia: a long-term consequence of hemolytic disease of the fetus and newborn?. Int. J. Ment. Health.

[R31] Hollister JM, Laing P, Mednick SA (1996). Rhesus incompatibility as a risk factor for schizophrenia in male adults. Arch. Gen. Psychiatry.

[R32] Hovatta I, Suvisaari J, Terwilliger JD, Ollikainen V, Arajarvi R, Juvonen H, Kokko-Sahin M-L, Vaisanen L, Mannila H, Lonnqvist J, Peltonen L (1999). A genomewide screen for schizophrenia genes in an isolated Finnish subpopulation, suggesting multiple susceptibility loci. Am. J. Hum. Genet.

[R33] Hsieh HJ, Palmer CGS, Harney S, Newton JL, Wordsworth P, Brown MA, Sinsheimer JS (2006a). The v-MFG test: Investigating maternal, offspring, and maternal-fetal genetic incompatibility effects on disease and viability. Genet. Epidemiol.

[R34] Hsieh HJ, Palmer CGS, Sinsheimer JS (2006b). Allowing for missing data at highly polymorphic genes when testing for maternal, offspring and maternal-fetal genotype incompatibility effects. Hum. Hered.

[R35] Hsieh HJ, Palmer CGS, Harney S, Chen H-W, Bauman L, Brown MA, Sinsheimer JS (2007). Using the maternal-fetal genotype incompatibility test to assess non-inherited maternal HLA-DRB1 antigen coding alleles as rheumatoid arthritis risk factors. BMC Proc.

[R36] Hultman CM, Sparen P, Takei N, Murray RM, Cnattingius S (1999). Prenatal and perinatal risk factors for schizophrenia, affective psychosis, and reactive psychosis of early onset: case-control study. Br. Med. J.

[R37] Insel B, Brown AS, Bresnahan MA, Schaefer CA, Susser ES (2005). Maternal-fetal blood incompatibility and the risk of schizophrenia in offspring. Schizophr. Res.

[R38] Kendell RE, McInneny K, Juszczak E, Bain M (2000). Obstetric complications and schizophrenia. Br. J. Psychiatry.

[R39] Kraepelin E (1919/1971). Dementia Praecox and Paraphrenia (R.M. Barclay, Trans).

[R40] Kraft P, Palmer C, Woodward JA, Turunen JA, Minassian S, Paunio T, Lonnqvist J, Peltonen L, Sinsheimer JS (2004). RHD maternal-fetal genotype incompatibility and schizophrenia: extending the MFG test to include multiple siblings and birth order. Eur. J. Hum. Genet.

[R41] Lange K (1991). SEARCH.

[R42] Lange K, Cantor R, Horvath S, Perola M, Sabatti C, Sinsheimer J, Sobel E (2001). Mendel Version 4.0: a complete package for the exact genetic analysis of discrete traits in pedigree and population data sets. Am. J. Hum. Genet.

[R43] Leung A, Chue P (2000). Sex differences in schizophrenia: a review of the literature. Acta Psychiatr. Scand.

[R44] Moises HW, Zoega T, Gottesman II (2002). The glial growth factors deficiency and synaptic destabilization hypothesis of schizophrenia. BMC Psychiatry.

[R45] Mollison PL (1993). Haemolytic Disease of the Fetus and Newborn.

[R46] Mueser K, McGurk S (2004). Schizophrenia. Lancet.

[R47] O'Callaghan E, Gibson T, Colohan HA, Buckley P, Walshe DG, Larkin C, Waddington JL (1992). Risk of schizophrenia in adults born after obstetric complications and their association with early onset of illness: a controlled study. Br. Med. J.

[R48] Palmer CGS, Turunen JA, Sinsheimer JS, Minassian S, Paunio T, Lonnqvist J, Peltonen L, Woodward JA (2002). RHD maternal-fetal genotype incompatibility increases schizophrenia susceptibility. Am. J. Hum. Genet.

[R49] Palmer CGS, Hsieh HJ, Reed EF, Lonnqvist J, Peltonen L, Woodward JA, Sinsheimer J (2006). HLA-B maternal-fetal genotype matching increases risk for schizophrenia. Am. J. Hum. Genet.

[R50] Paunio T, Ekelund J, Varilo T, Parker A, Hovatta I, Turunen JA, Rinard K, Foti A, Terwilliger JD, Juvonen H, Suvisaari J, Arajarvi R, Suokas J, Partonen T, Lonnqvist J, Meyer J, Peltonen L (2001). Genome-wide scan in a nationwide study sample of schizophrenia families in Finland reveals susceptibility loci on chromosomes 2q and 5q. Hum. Mol. Genet.

[R51] Plevyak MP, Carr SR (2001). Rhesus isoimmunization. Med. Health R.I.

[R52] Preti A, Cardascia L, Zen T, Marchetti M, Favaretto G, Miotto P (2000). Risk for obstetric complications and schizophrenia. Psychiatry Res.

[R53] Renkonen KO, Timonen S (1967). Factors influencing the immunization of Rh-negative mothers. J. Med. Genet.

[R54] Sacker A, Done JD, Crow TJ, Golding J (1995). Antecedents of schizophrenia and affective illness: obstetric complications. Br. J. Psychiatry.

[R55] Salem JE, Kring AM (1998). The role of gender differences in the reduction of etiologic heterogeneity in schizophrenia. Clin. Psychol. Rev.

[R56] Sinsheimer JS, Palmer CGS, Woodward JA (2003). Detecting genotype combinations that increase risk for disease: the maternal-fetal genotype incompatibility test. Genet. Epidemiol.

[R57] Sullivan PF, Kendler KS, Neale MC (2003). Schizophrenia as a complex trait. Arch. Gen. Psychiatry.

[R58] Thorup A, Waltoft BL, Pedersen CB, Mortensen PB, Nordentoft M (2007). Young males have a higher risk of developing schizophrenia: a Danish register study. Psychol. Med.

[R59] Ulm B, Svolba G, Ulm MR, Bernaschek G, Panzer S (1999). Male fetuses are particularly affected by maternal alloimmunization to D antigen. Transfus.

[R60] Verdoux H, Bourgeois M (1993). A comparative study of obstetric history in schizophrenics, bipolar patients, and normal subjects. Schizophr. Res.

[R61] Verdoux H, Geddes JR, Takei N, Lawrie SM, Bovet P, Eagles JM, Heun R, McCreadie R, McNeil TF, O'Callaghan E, Stober G, Willinger MU, Wright P, Murray RM (1997). Obstetric complications and age at onset in schizophrenia: an international collaborative meta-analysis of individual patient data. Am. J. Psychiatry.

[R62] Wagner FF, Flegel WA (2000). RHD gene deletions occurred in the Rhesus box. Blood.

[R63] Westhoff CM (2007). Rh complexities: serology and DNA genotyping. Transfus.

[R64] Woodrow JC, Donohoe WTA (1968). Rh-immunization by pregnancy: results of a survey and their relevance to prophylactic therapy. Br. Med. J.

